# Norovirus GII.3[P12] Outbreak Associated with the Drinking Water Supply in a Rural Area in Galicia, Spain, 2021

**DOI:** 10.1128/spectrum.01048-22

**Published:** 2022-07-14

**Authors:** Camille Jacqueline, Manuel del Valle Arrojo, Paloma Bellver Moreira, Maria Asunción Rodríguez Feijóo, Maria Cabrerizo, Maria Dolores Fernandez-Garcia

**Affiliations:** a Centro Nacional de Microbiologia, Instituto de Salud Carlos III, Majadahonda, Madrid, Spain; b European Public Health Microbiology Training Programme (EUPHEM), European Centre for Disease Prevention and Control (ECDC), Stockholm, Sweden; c Xefatura territorial de sanidade da Coruña, Sección de epidemioloxía, Coruña, Spain; d Laboratorio de Salud Pública de Galicia, Lugo, Spain; e Servicio de Microbiología, Complejo Hospitalario Universitario A Coruña, Coruña, Spain; f Instituto de Salud Carlos III, CIBERESP, Madrid, Spain; Emory University School of Medicine

**Keywords:** genotyping, drinking water, noroviruses, outbreak, transmissible gastroenteritis virus, waterborne pathogens

## Abstract

On 30 September 2021, the city council of Muxia, Spain (population of 4,564 inhabitants), reported an unusual increase of patients with acute gastroenteritis (AGE). Because geographically widespread villages belonging to the same water supply were affected, a waterborne outbreak was suspected. Overall, 115 probable cases were ascertained during epidemiological investigations carried out by the local health authority (attack rate, 5.7%); the age range was 0 to 92 years, and 54% were female. The main symptoms were vomiting (78.1%) and diarrhea (67.5%). Primary cases peaked on 29 September and subsided on 1 October, compatible with a point-source outbreak followed by possible secondary cases until 7 October. We conducted an unmatched case-control study using phone surveys. The case-control study included 62 cases and 46 controls. Univariate analysis showed that cases had a higher exposure to tap water through direct consumption (odds ratio [OR] = 86; 95% confidence interval [CI], 18 to 409) or vegetable washing (OR = 27; 95% CI, 7 to 98). Norovirus GII was detected in two terminal points of the water supply system, and 14 cases were laboratory confirmed after detection of GII in stool samples. A unique genotype (GII.3[P12]) was identified in stool samples. On 1 October, a tap water ban was put in place and the water was purged and chlorinated. The rapid increase in the number of cases and its decline after implementing control measures suggested a waterborne point-source outbreak among the residents of Muxia sharing the same water distribution system.

**IMPORTANCE** Noroviruses are likely to be underrecognized in most suspected waterborne outbreaks. Therefore, effective norovirus detection and the early recognition of water as a possible source of infection are important to reduce morbidity as appropriate steps are taken to control the source. In our study, we combined epidemiological, environmental, and microbiological investigations to demonstrate that it was a waterborne outbreak caused by norovirus. Metagenomic sequencing in one norovirus-positive stool sample confirmed norovirus etiology and the absence of other potential pathogens. Detection of fecal indicator bacteria and the fact that the drinking water was not chlorinated suggest a breakdown in chlorination as the cause of the outbreak. This outbreak investigation also demonstrated the importance of timely communication to the public about the risk linked to tap water consumption.

## INTRODUCTION

Despite advances in water treatment technologies, drinking water outbreaks caused by microbial contamination are still frequent in high-income countries and may acutely infect many people, simultaneously posing a significant risk to public health (1). Drinking water supply systems are the most important source of water-related infectious diseases in the pan-European region (2). Between 2000 and 2013 in the World Health Organization (WHO) European Region, viral gastroenteritis accounted for the highest total numbers of water-related disease outbreaks (3).

Noroviruses (NoVs) are the predominant cause of all nonbacterial acute gastroenteritis (AGE) worldwide (4). Disease occurs across all age groups. The primary route of transmission is fecal-oral either by ingestion of contaminated water or food or by direct person-to-person transmission (4). Noroviruses are highly resistant to high levels of chlorine, heat, cold, acidic pH, and organic solvents, which allows their survival for long periods in the environment (5). Their high infectiousness and efficient transmission rely also on the short duration of immune protection, the low infectious dose (10 to 100 viral particles), high viral loads shed by infected individuals, and the frequency of asymptomatic infections (4). These viral properties make norovirus a frequent cause of waterborne outbreaks.

In waterborne outbreaks, the assessment of NoV etiology is based on the detection and typing of viruses from stool and water samples in association with epidemiologic evidence. Norovirus typing is performed through genogroup and genotype assignments. Noroviruses are classified into 10 genogroups (G), of which GI and GII are the most prevalent in humans, and ≥48 genotypes (6). Norovirus genotyping strategy considers the genotype encoding the major capsid protein VP1 (G-type) and the genotype encoding the polymerase region (P-type) (6).

The early recognition of water as a possible source of norovirus infection can reduce morbidity and mortality as appropriate steps are taken to identify and control the source. However, unlike for bacteria, there is not a legislative requirement to routinely monitor water supplies for the presence of norovirus. Therefore, prompt identification of a norovirus-mediated waterborne outbreak is not feasible until symptomatic cases arise and are caught in syndromic surveillance. Moreover, given that infected cases are largely asymptomatic and that norovirus causes a common illness, usually large numbers of symptomatic cases are required for health care workers and authorities to recognize water as a possible source of infection (7). Besides, norovirus may not always be detected in the water supply system for a number of reasons including (i) the amount of time that may have elapsed between the contamination event and exposure of cases to the contaminant, (ii) the brief persistence of the virus in the water, and (iii) the use of inappropriate detection methods together with insufficient sample volume (2). Consequently, noroviruses are not directly detected in water samples in most suspected norovirus waterborne outbreaks. In Spain, waterborne outbreaks associated with norovirus infection have been rarely reported in the literature, with only 6 outbreak investigations published since 1995 (8–13). These outbreaks were associated with sources of infection such as bottled mineral water, spring water, or municipal drinking water systems, but norovirus was directly detected in water samples in only half of these outbreaks.

### Outbreak detection.

On 30 September 2021, the Provincial Health Office (PHO) (A Coruña, Galicia, Spain) received a phone call from the city council of Muxia related to an increase of AGE in Muxia, a small seaside city (population of approximatively 5,000 persons) in a rural area. The increase of AGE was noticed from 28 September but had not been reported by the health center of Muxia nor the hospital of the region (Hospital Virxe da Xunquira, Cee, Galicia). The PHO contacted the health center and the emergency service of the hospital and confirmed the increase in AGE compared to other years at the same period. Because of the wide geographical distribution, a waterborne outbreak was suspected, and an investigation was immediately undertaken to assess the extent of the outbreak, to identify the etiological agent, and to implement control measures.

## RESULTS

### Epidemiological study. (i) Description.

A total of 115 probable cases were identified between 27 September and 7 October. Only one case (male, 4 months old) required a 1-day hospitalization. Fourteen patients met the definition of confirmed case. The observed attack rate in the population supplied by the water distribution system of Muxia was 5.7% (115/2,022). The most common symptom was vomiting (*n* = 89, 78.1%), followed by diarrhea (*n* = 77, 67.5%), nausea (*n* = 62, 54%), abdominal pain (*n* = 60, 52.7%), and headache (*n* = 45, 39.1%). The average age was 37.2 years (range, 0 to 92 years), and 54.4% were female.

**FIG 1:**
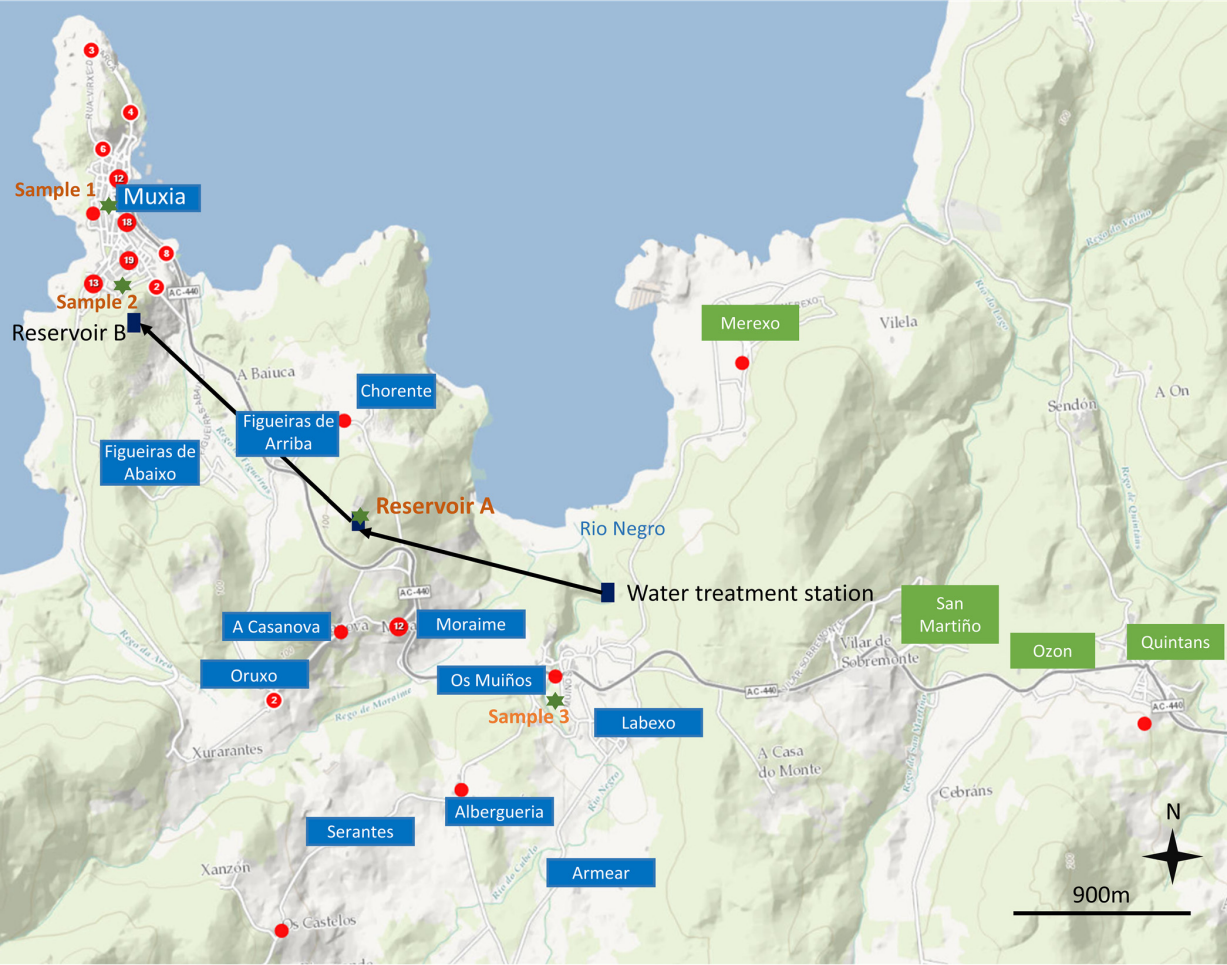
Representation of the drinking water supply system of Muxia and distribution of cases in the affected area. Each red circle represents a probable case or a number of cases as written in white in the circle. Cities belonging to the water supply system of Muxia are shown in blue squares, and cities from which controls were selected are shown in green. The locations of the water samples taken in this study are shown with green stars. We used the software epiInfo v7.2.5.0 and the background map was obtained from https://services.arcgisonline.com/ArcGIS/rest/services/World_Street_Map/MapServer.

**FIG 2:**
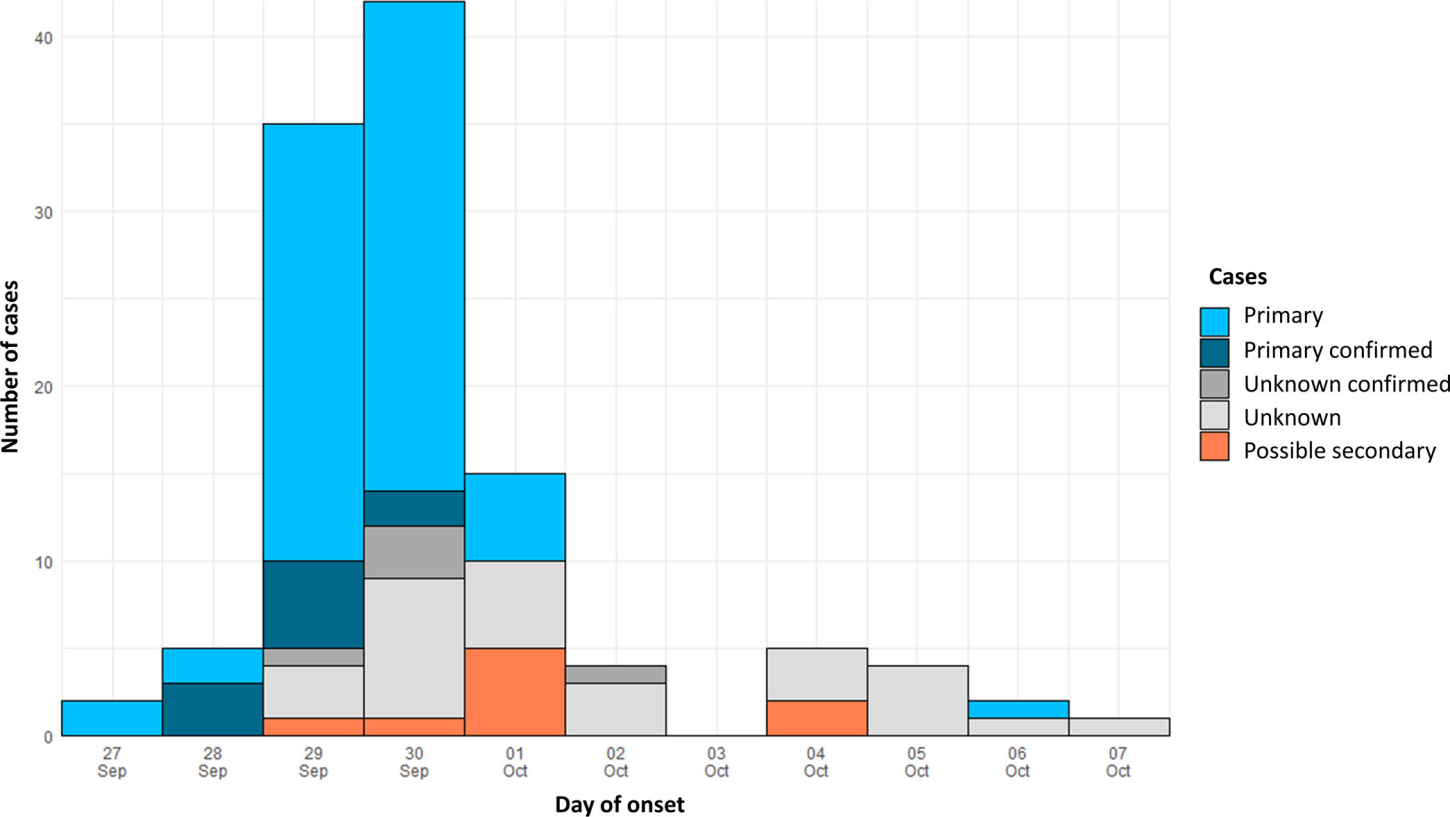
Epidemic curve showing date of illness onset for probable cases (*n* = 115) in the outbreak, Spain, 2021. Primary cases are shown in light blue and, when they were laboratory confirmed, in dark blue, while possible secondary cases (defined as cases in Muxia reporting contact with another case in the 3 days before symptom onset) are shown in orange. Cases with unknown status are shown in light gray and, when laboratory confirmed, in dark gray.

Most cases (*n* = 87) occurred in Muxia, and 27 occurred in several surrounding localities (Fig. 1). This widespread distribution corresponded to the water distribution system of Muxia (Fig. 1) and therefore supported the hypothesis of a waterborne outbreak. Figure 2 shows the distribution of cases over time. The first case was reported on 27 September, but it is only from 28 September that a growing number of inhabitants reported illness, until 29 September. Illness reports peaked on 30 September with 46 inhabitants affected. The number of primary cases subsided after 1 October, and the last case was reported on 7 October. Cases of unknown status reported after 1 October might correspond to secondary cases (Fig. 2). We hypothesize that the case on 27 September was probably unrelated to the outbreak as cases were not reported for the next 24 h. The rapid increase of cases and its decline after the implementation of restrictions on tap water consumption suggested that the etiologic agent responsible for the outbreak had a short period of incubation and reinforced the hypothesis of a waterborne point-source outbreak among the residents of Muxia sharing the same water distribution system.

### (ii) Analysis: case-control study.

The case-control study recruited 62 (69%) probable primary cases and 46 (77%) controls who completed the questionnaire. In the univariate analysis (Table 1), two exposures were associated with illness, (i) tap water consumption (odds ratio [OR] = 8.4; 95% confidence interval [CI], 2 to 30) and (ii) washing of vegetables with tap water (OR = 27.8; 95% CI, 7 to 98). These two variables combined, i.e., exposure to the water distribution system, were associated with an OR of 86.4 (95% CI, 18 to 409). Although several exposures were associated with gastrointestinal illness in the univariate analysis, 96.8% of the cases were exposed to the water distribution system compared to 26.1% of the controls (who visited relatives or restaurants in the city of Muxia), suggesting that water from the distribution system was the source of the infection. The odds of being a case decreased statistically significantly with the exposure to water from another distribution system (*P* < 0.001) or to spring water (OR, 0.34; 95% CI, 0.14 to 0.84). Consumption of water from a well, of bottled water, and of mollusks did not significantly impact the odds of being a case.

**TABLE 1:** Probable cases and controls according to frequency of exposure to selected factors, Muxia municipality, 27 September to 7 October 2021^*a*^

Exposure	% cases (*n* = 62)	% controls (*n* = 46)	OR (95% CI)	*P* value
Water supply from Muxía	96.8	26.1	86.42 (18.26–409.0)	
Tap water	41.9	7.9	8.43 (2.34–30.38)	
Washing vegetables with tap water	93	32.3	27.83 (7.85–98.58)	
Tap water from another water supply	0	77.8	NC	0.0001
Water from a well	8.8	8.1	1.09 (0.24–4.86)	
Spring water	24.1	48.5	0.34 (0.14–0.84)	
Bottled water	80	42.4	5.43 (0.55–54.0)	
Consumption of mollusks	0	2.7	NC	0.378

a CI, confidence interval; NC, not calculable.

### Environmental study findings.

Analysis by the Public Health Laboratory of Galicia (PHLG) found that the water in the public fountain (sample 1) contained 0.0 ppm of free chlorine, 36 MPN (most probable number) of Escherichia coli, >200 MPN of total coliforms, and 3 CFU/100 mL of Clostridium perfringens. The water from the high school (sample 2) contained 0.0 ppm of free chlorine and 15 CFU/100 mL of Clostridium perfringens. These parameters showed concentrations outside acceptable parameter ranges (0.2 to 5 ppm for free chlorine, 0 MPN for Escherichia coli, 0 MPN for total coliforms, and 0 CFU/100 mL for Clostridium perfringens). Samples were also found to be positive for norovirus GII, but sequence-based typing on water samples could not be obtained. After analyses, the private laboratory found that the water in the middle school (sample 3) and Reservoir A contained 28 and 13 CFU/100 mL of Clostridium perfringens, respectively.

### Clinical sample investigation.

**FIG 3:**
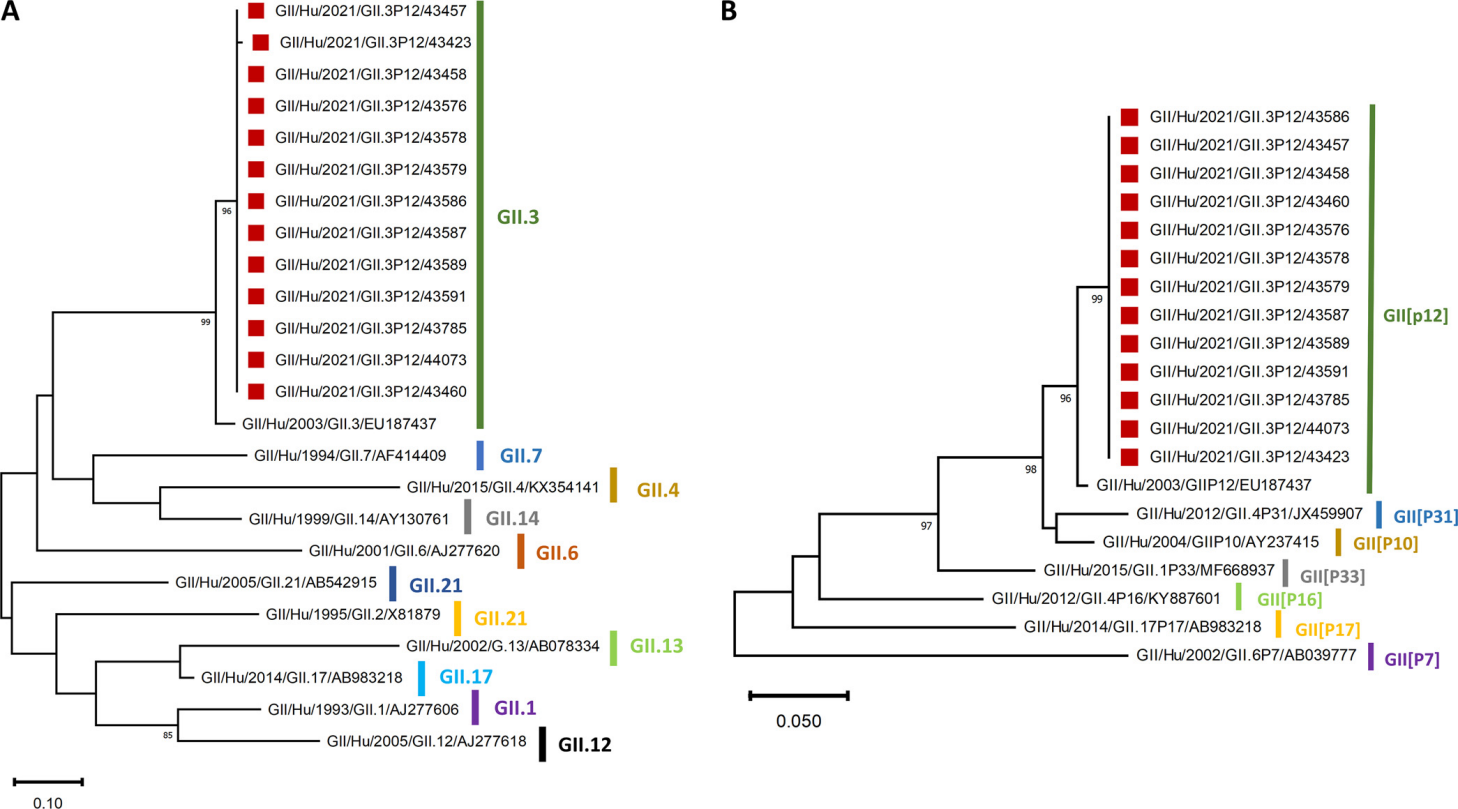
Phylogenetic trees based on the 229-bp nucleic acid sequences of the norovirus capsid region (A) and the 243-bp nucleic acid sequences of the polymerase region (B). Bootstrap values above 80 are represented. Red squares indicate the strains collected in the outbreak. Abbreviations of virus names indicate genogroup/sample origin (Hu, human)/year of isolation/genotype/identification number or GenBank accession number for reference sequences.

Using immunochromatography, the Hospital of A Coruña (CHUAC) identified two cases as positive for norovirus GII from the 12 probable cases that were analyzed (Table 2). Coprocultures for bacterial identification were negative. Then, 15 samples were sent to the National Center for Microbiology (CNM), and 13 (87.5%) could be amplified and sequenced. All 13 sequences were classified as GII.3[P12] strains. All patient samples had 100% sequence identity, showing the high clonality and confirming that they belonged to the same outbreak. These associations were confirmed by high bootstrap values in the phylogenetic analysis (Fig. 3). Because norovirus diversity can be underestimated when using conventional molecular genotyping analysis, such as Sanger sequencing of only a limited part of the genome (14), we also used metagenomic sequencing to compare our result with the ones obtained with the Sanger method. The identification of a unique genotype, GII.3[P12], was confirmed by the sequencing of one outbreak strain using metagenomics. We observed no evidence of new recombination events (data not shown).

**TABLE 2:** Human and water samples used for microbiological and environmental investigations^*a*^

Sample origin and no.	Sex	Age (yr)	Onset of symptoms (mo/day/yr, time)	Date of sample collection (mo/day/yr)	Time (days) between onset and collection	Result from laboratory:		
						CHUAC	CNM	PHLG
Human								
1	F	57	9/29/21, 9:00 p.m.	10/1/21	2	GII^+^	GII.3[P12]	
2	F	34	9/28/21, 7:30 p.m.	10/1/21	3	Negative	GII.3[P12]	
3	F	15	9/29/21, 12:00 p.m.	10/1/21	2	Not tested	GII.3[P12]	
4	M	7	9/30/21, 5:00 p.m.	10/4/21	4	GII^+^	Not tested	
5	F	25	9/28/21, 11:30 p.m.	10/1/21	3	Negative	GII.3[P12]	
6	M	16	9/30/21, 3:00 a.m.	10/5/21	5	Not tested	Not tested	
7	M	40	9/29/21, 12:00 p.m.	10/1/21	2	Negative	GII.3[P12]	
8	M	66	9/29/21, 11:00 p.m.	10/1/21	2	Ad^+^	GII.3[P12]	
9	F	86	9/30/21, 12:00 a.m.	10/1/21	1	Not tested	Negative	
10	F	62	9/28/21, 10:00 p.m.	10/1/21	3	Negative	GII.3[P12]	
11	F	36	9/30/21, 12:00 a.m.	10/1/21	1	Negative	GII.3[P12]	
12	M	7	9/29/21, 3:00 a.m.	10/1/21	2	Not tested	GII.3[P12]	
13	M	43	9/30/21, 10:00 a.m.	10/1/21	1	Negative	GII.3[P12]	
14	M	16	9/29/21, 11:00 p.m.	10/1/21	2	Not tested	GII.3[P12]	
15	M	73	10/5/21, 12:00 p.m.	10/11/21	6	Negative	Not tested	
16	M	80	9/30/21, 12:00 p.m.	10/6/21	6	Not tested	GII.3[P12]	
17	F	85	10/2/21, 10:00 p.m.	10/5/21	3	Negative	GII.3[P12]	
18	M	0	9/29/21, 6:00 p.m.	10/1/21	2	Negative	Not tested	
19	F	34	10/1/2021, 11:45 a.m.	10/4/21	3	Not tested	Negative	
Water								
1				9/30/21				GII^+^
2				9/30/21				GII^+^

a F, female; M, male; GII^+^, positive for norovirus GII; Ad^+^, positive for adenovirus; CHUAC, Hospital of A Coruña; CNM, National Center for Microbiology; PHLG, Public Health Laboratory of Galicia.

### Control measures and communication with the public.

On 30 September, the city council of Muxia issued a recommendation to avoid drinking tap water until microbiological results were obtained. The communication was published at the city hall, in a municipal mobile application, and in a press release. On 1 October, following the results from the environmental analyses revealing the presence of norovirus in the water, the distribution of water was stopped. This order was relayed by the local press on the same day. The municipality provided bottled water to the inhabitants of Muxia as an alternative. Water decontamination started on the afternoon of the same day following national recommendations and included hyperchlorination and purges of the network. Water distribution was reinitialized the 2 October for utilities (shower, toilets, plant watering, etc.). On 4 October, further controls on the same points of the water distribution system showed normal levels of bacterial indicators. The municipality communicated through the mobile application the results of the analyses, and the tap water ban was lifted. Even though the number of reported primary cases dramatically decreased after 1 October, active surveillance of new AGE cases was implemented until 8 October.

## DISCUSSION

We report epidemiological, environmental, and microbiological investigations of a large-scale gastroenteritis outbreak associated with norovirus GII.3[P12] contamination of the drinking water supply. Norovirus is commonly transmitted via direct contact with infected persons (person to person), consumption of contaminated food and water, or contact with a contaminated environment (4). Given the typical point-source outbreak curve (Fig. 2), person-to-person transmission was not likely to be the primary cause of transmission in this outbreak even though sporadic cases after 1 October (when the last primary case was reported) could be associated with secondary transmission. It highlights the importance even in the context of waterborne outbreaks to communicate and emphasize proper hygiene practices for people in contact with symptomatic patients to prevent secondary transmission within households (2). According to the scientific literature, waterborne outbreaks of norovirus have been often associated with high attack rates (8, 15, 16). For example, another study in Spain showed an attack rate of 64.1% for a norovirus waterborne outbreak in a holiday camp (8). Therefore, the attack rate of 5.7% in the population supplied by the distribution system of Muxia was relatively low. We hypothesize that the number of cases might have been largely underestimated as milder cases and adults are less likely to seek medical attention especially in rural areas where distances and commuting times to access health care centers are longer.

Chlorination is normally done at the exit of the water reservoir to allow sufficient time for activation before distribution. Environmental investigation confirmed that the free chlorine levels at two terminal points of the water distribution system of Muxia were 0.0 ppm and that fecal indicators like E. coli and C. perfringens were present in drinking water. A study showed that drinking water outbreaks caused by microbial contamination between 2003 and 2013 in developed countries were attributed to failures in infrastructure and distribution networks or institutional practices such as poor operational and maintenance practices (aged infrastructure, inadequate monitoring, and negligence) (1). In addition, heavy rainfall together with flooding is associated with increased risk of viral contamination in the drinking water as it can trigger discharges of raw sewage or runoff animal manure (2, 17). During the weeks before the outbreak, no failures in the water pipe network or falls in the water pressure or heavy rainfalls were reported (18). Given that the water treatment station of Muxia treats water from the river (Rio Negro), fecal spill into the river was also suspected. However, due to the high environmental stability of norovirus in water (it may persist for up to 2 months in river water [19]), an event of fecal contamination in the Rio Negro was not necessarily contemporaneous with the outbreak, and therefore, the source or the time of the event responsible for the contamination could not be identified. Moreover, outbreaks associated with massive fecal contamination are often characterized by more than one norovirus genotype and usually caused by multiple viruses and etiologic pathogens (20, 21). In the outbreak presented here, a unique genotype of norovirus was found, even though samples were tested for GI and GII genotypes, and no bacterial infection was detected. Metagenomic sequencing confirmed the sole presence of norovirus in a stool sample, providing supporting evidence that the origin of the outbreak might not be fecal discharge into the river. Similarly, other studies have described the involvement of a single norovirus genotype in waterborne outbreaks (8, 22).

Interestingly, we identified a norovirus GII strain as the causative agent, rather than GI strains, which are predominant in waterborne norovirus outbreaks (15, 23, 24). The reason for GI association with waterborne outbreaks is not known, but it has been hypothesized that GI strains might be more stable in water than GII strains (7, 24). Nonetheless, GII strains have also been implicated in waterborne norovirus outbreaks (7, 8, 22, 25). In this study, all the 10 stool samples that were negative by the immunochromatography method were positive for GII by the PCR method. This is consistent with previous studies showing low sensitivity (between 23 and 59%) for norovirus immunochromatographic methods (26, 27) and highlights the added value of using PCR for norovirus detection during outbreaks instead of immunochromatography.

We identified genotype GII.3[P12] in 13 out of 15 stool samples with identical genomic sequences between patients, suggesting a common source of infection. To the best of our knowledge, this was the first norovirus GII.3[P12] outbreak detected in Spain. Currently, genotype GII.4 is the most common cause of norovirus infection worldwide and in Spain (28, 29). However, non-GII.4 strains such as GII.2, GII.3, and GII.6 viruses are also commonly implicated in AGE cases (29). Studies on the evolution of GII.3[P12] are scarce, but it was recently suggested that this specific genotype was initially restricted to Asia and the first case was indeed reported in Japan in 2003 (30). Surveillance data show that GII.3[P12] first started to circulate in Spain in the last quarter of 2020 (2 cases) and in the first quarter of 2021 (10 cases) in the region of Valencia in children younger than 5 years of age (norovirus.org). Therefore, even though GII.3[P12] was circulating in Spain before 2021 (29, 31), it remains to be seen if the transmission of this genotype will be responsible for other outbreaks and establish as a new endemic norovirus genotype. Although norovirus-mediated outbreaks are generally associated with winter conditions, the present study also shows that norovirus should be considered when determining the etiology of an outbreak even during interseasonal periods.

There are some limitations to this study. First, it is known that up to 32% of norovirus infections may be asymptomatic (32); therefore, the number of cases might be largely underestimated. In this situation, controls might have been wrongly identified on the absence of symptoms. Second, the majority of the cases did not send feces samples for further characterization, which limits the genetic characterization of the outbreak. Third, norovirus genotyping from water samples was unsuccessful. This could be due to RNA degradation during sample transportation or due to low virus levels in water samples. Indeed, genotyping PCRs are generally less sensitive than real-time PCRs (33), and our experience has shown that reverse transcription-PCR (RT-PCR) has a limit of detection between 660 and 11,000 norovirus genome copies per L of water. Regarding this, it should be considered that the water samples were taken on 30 September at the peak of the outbreak when the virus could be present at lower concentrations if it was a transient contamination. Another limitation was that, although stool samples were also tested for rotavirus, adenovirus, and fecal bacteria, we cannot exclude the chance that other pathogens were involved in the etiology of the outbreak as water and stool samples were not tested for protozoa or parasites. However, because most patients showed mild symptoms and only one patient (male, 4 months old) was hospitalized, coinfection is unlikely to have occurred. Finally, epidemiological standardized questionnaires are routinely used when investigating waterborne norovirus outbreaks, limiting recall bias especially in controls, and the attribution of the wrong status of exposure.

The outbreak had an impact in terms of the number of people who missed school or work activities as a consequence of their illness. In fact, the number of absences in school strongly increased after 30 September even though this number does not necessarily reflect the number of children with gastroenteritis. The public health implications could have been much more serious had this outbreak been caused by a more virulent pathogen than norovirus, which is generally a mild and self-limiting illness.

In conclusion, results from epidemiological, environmental and microbiological investigations support norovirus-contaminated drinking water as the source of this outbreak. Detection of fecal indicator bacteria and the fact that the drinking water in Muxia was not chlorinated suggest suboptimal treatment performance like a breakdown in chlorination as the cause of the outbreak. This outbreak investigation demonstrated the importance of timely communication with the public about the risk linked to tap water consumption. Development of a water safety plan following methods given by the WHO is recommended for municipalities’ water as an effective way to ensure water safety (34). In addition, enhancement of the laboratory investigation of waterborne outbreaks in the country with promotion of diagnostic testing for viruses in water samples and implementation of a collaboration protocol between local authorities and reference laboratories, which would clearly define when water samples should be collected and how they should be stored and transferred for virological testing, even in the absence of bacterial indicators of fecal contamination in water samples, are essential. Finally, efforts should be made to identify the source of the contamination to prevent further outbreaks in the region, and particular attention should be given to the monitoring of fecal indicators at different points of the water distribution system of Muxia.

## MATERIALS AND METHODS

### Epidemiological studies.

Probable cases were defined as a person experiencing symptoms of vomiting and/or diarrhea who lived in the municipality of Muxia between 28 September and 7 October 2021. Confirmed cases were defined as probable cases that tested positive for the presence of norovirus GII in the stool. We also distinguished primary cases, who did not report contact with a probable case in the 3 days before onset of symptoms, from possible secondary cases, who reported such contact but could also be primary cases as they lived in Muxia and were potentially exposed to tap water. In addition, for some cases, we were unable to establish the presence or the absence of contact with a probable case and they were therefore designated unknown cases. To increase the power of epidemiological investigation, we focused on probable cases for the descriptive and analytical analyses. An unmatched case-control study was conducted to test hypotheses on the source of infection. Possible secondary cases and cases with unknown status were excluded. One control per case was selected from the Muxia population register but from villages that did not belong to the water distribution system of Muxia (Fig. 1). Controls were included if they had consumed tap water from any water distribution system in the 3 days before 28 September and until 30 September. Controls with AGE 15 days before were excluded. Probable cases and controls were interviewed by phone using a standardized questionnaire that included items on tap, well, or spring water consumption; washing food with water; consumption of mollusks; attendance at a party or a dinner; and contact with a person with AGE 3 days before symptom onset. Odds ratios (ORs) and 95% confidence intervals were calculated for different exposures of interest. In the case that we could not calculate the OR, we measured the *P* value (Fisher exact test). Statistical analyses were realized using the software SPSS v.19.

### Environmental studies.

In the municipality of Muxia, the principal water distribution system supplies the city center and some suburbs (Fig. 1). On 30 September at 2 p.m., environmental officers collected water in a public fountain (sample 1) and in a high school (sample 2) of Muxia (Fig. 1). The samples were further analyzed, at the PHLG, to establish the conformity of organoleptic and physicochemical values to legislation standards for drinking water. In addition, chlorine concentration was measured and the presence of bacterial indicators for fecal contaminations was investigated according to the ISO standard 9308-2. Clostridium perfringens was detected by culture on tryptose-sulfite-cycloserine agar medium. Escherichia coli and total coliforms were detected using a Colilert substrate assay with Quanty-Tray (Idexx Laboratories, USA).

For norovirus analysis, water samples were analyzed with a protocol based on the ISO standards 15216-1 and -2 (35). The virus adsorption-elution technique was used to concentrate the virus. Briefly, 1 L of each water sample was vacuum filtered in a 0.4-μm polycarbonate filter (36). Two controls were included and processed similarly: (i) water inoculated with a titrated mengovirus to ensure the RNA extraction efficiency (KMG kit, ceeramTOOLS) and (ii) inoculated water to serve as a negative extraction control. The adsorbed viruses from the membrane were then eluted using Tris-glycine-beef extract (TGBE) buffer (100 mM Tris-HCl, 50 mM glycine, 1% beef extract, pH 9.5). Viruses were further concentrated by ultrafiltration with Amicon 100,000-molecular-weight-cutoff (MWCO) columns to reach a final volume of concentrated water equal to 0.5 mL. The totality of the concentrated water was used to extract RNA using the MagMAX viral RNA isolation kit (ThermoFisher) according to the manufacturer’s instructions, and the final elution was made in 100 μL. Finally, qualitative detection was performed using RT-PCR kits for detection of norovirus GI (KNVGI) and GII (KNVGII; ceeramTOOLS; bioMérieux) according to the manufacturer’s specifications. RNA extracts (pure and 10-fold-diluted RNA) were analyzed in duplicate for norovirus and once (pure and 10-fold-diluted RNA) for mengovirus. Spiked samples were included to estimate the practical limit of detection and consisted of norovirus genogroup I and II certified and titrated reference material (Centre for Environment, Fisheries and Aquaculture Science [CEFAS], Public Health England [PHE]). Results were considered valid when extraction efficiency was equal to or greater than 1%.

In addition, a private laboratory was contracted by the water supply manager to sample and analyze waters in other points of the system: the middle school in Muiños (sample 3) and reservoir A (Fig. 1).

### Clinical sample investigation.

After the report of the city council of Muxia, the health center of Muxia and the emergency service of the hospital of Virxe da Xunquira were contacted by the PHO. They were asked to report the cases of AGE and to transmit feces samples for patients corresponding to the case definition. Nineteen stool samples from patients who reported illness between 27 September and 7 October were obtained. Most of the samples were collected in the 3 days after the onset of symptoms. Twelve stool samples were analyzed by the CHUAC. Coprocultures were carried out on several selective media (MacConkey agar with and without sorbitol, blood agar, *Yersinia* agar, Salmonella-*Shigella* agar, Campylobacter agar, and *Clostridium* agar) and one enrichment medium (selenite broth). A rapid immunochromatography test (Certest; Biotec, S.L.) was used for detection of rotavirus, adenovirus, and norovirus GI and GII. Fifteen stool samples (including 9 previously tested by CHUAC) were sent to the CNM. Dual polymerase-capsid genotyping was performed by using one-step conventional RT-PCR protocol for GI and GII (37) and an online tool (http://www.rivm.nl/mpf/norovirus/typingtool). Complete genome sequence was further obtained from one randomly selected outbreak strain. Briefly, stool samples were diluted 1:10 with phosphate-buffered saline (PBS), vortexed for 30 s, and clarified by centrifugation at 4,000 × *g* for 10 min. Viral RNA was extracted using the Quick RNA viral kit (Zymo). Sample library preparation was conducted using the NEBNext Ultra RNA library prep kit for Illumina with NEBNext Multiplex Oligos for Illumina Set 3 (New England BioLabs Inc., USA). Libraries were sequenced on an Illumina NextSeq500 sequencer (300 cycles). The resulting data were analyzed using the viralrecon pipeline (https://github.com/nf-core/viralrecon). Reads assigned to the host’s genome were removed for analyses. SPAdes v3.14.0 (3) in metaSPAdes mode was used to perform a *de novo* assembly of nonhost reads. Phylogenetic trees were constructed by the neighbor-joining method with 2,000 bootstrap replicates using Molecular Evolutionary Genetic Analysis (MEGA4) software. Bootscanning analysis was also carried out using Simplot v3.5.1.

### Data availability.

Sequences were submitted to GenBank under the accession numbers OM190423 to OM190435. The whole-genome sequence was submitted under accession number OM884056.
